# Exploring the Anthropometric, Cardiorespiratory, and Haematological Determinants of Marathon Performance

**DOI:** 10.3389/fphys.2021.693733

**Published:** 2021-09-03

**Authors:** Georgios A. Christou, Efstathios D. Pagourelias, Asterios P. Deligiannis, Evangelia J. Kouidi

**Affiliations:** Laboratory of Sports Medicine, Sports Medicine Division, Aristotle University of Thessaloniki, Thessaloniki, Greece

**Keywords:** marathon race, body fat percentage, echocardiography, cardiopulmonary exercise testing, haemoglobin

## Abstract

**Aim:**

We aimed to investigate the main anthropometric, cardiorespiratory and haematological factors that can determine marathon race performance in marathon runners.

**Methods:**

Forty-five marathon runners (36 males, age: 42 ± 10 years) were examined during the training period for a marathon race. Assessment of training characteristics, anthropometric measurements, including height, body weight (*n* = 45) and body fat percentage (BF%) (*n* = 33), echocardiographic study (*n* = 45), cardiopulmonary exercise testing using treadmill ergometer (*n* = 33) and blood test (*n* = 24) were performed. We evaluated the relationships of these measurements with the personal best marathon race time (MRT) within a time frame of one year before or after the evaluation of each athlete.

**Results:**

The training age regarding long-distance running was 9 ± 7 years. Training volume was 70 (50–175) km/week. MRT was 4:02:53 ± 00:50:20 h. The MRT was positively associated with BF% (*r* = 0.587, *p* = 0.001). Among echocardiographic parameters, MRT correlated negatively with right ventricular end-diastolic area (RVEDA) (*r* = −0.716, *p* < 0.001). RVEDA was the only independent echocardiographic predictor of MRT. With regard to respiratory parameters, MRT correlated negatively with maximum minute ventilation indexed to body surface area (VEmax/BSA) (*r* = −0.509, *p* = 0.003). Among parameters of blood test, MRT correlated negatively with haemoglobin concentration (*r* = −0.471, *p* = 0.027) and estimated haemoglobin mass (Hbmass) (*r* = −0.680, *p* = 0.002). After performing multivariate linear regression analysis with MRT as dependent variable and BF% (standardised β = 0.501, *p* = 0.021), RVEDA (standardised β = −0.633, *p* = 0.003), VEmax/BSA (standardised β = 0.266, *p* = 0.303) and Hbmass (standardised β = −0.308, *p* = 0.066) as independent variables, only BF% and RVEDA were significant independent predictors of MRT (adjusted R^2^ = 0.796, *p* < 0.001 for the model).

**Conclusions:**

The main physiological determinants of better marathon performance appear to be low BF% and RV enlargement. Upregulation of both maximum minute ventilation during exercise and haemoglobin mass may have a weaker effect to enhance marathon performance.

**Clinical Trial Registration:**

www.ClinicalTrials.gov, identifier NCT04738877.

## Introduction

Long-time endurance exercise training leads to cardiovascular, respiratory, haematological and neuromuscular adaptations. Specifically, endurance athletes are characterised by cardiac chamber enlargement along with improved ventricular diastolic function, enhanced ventilation during exercise, expansion of both plasma and erythrocyte volumes, increased skeletal muscle capillary density, attenuated decline in motor unit conduction velocity during sustained contractions of skeletal muscles and greater number and volume of skeletal muscle mitochondria (Hoppeler et al., 1973; Ingjer, 1979; Convertino, 1991; Layton et al., 2011; Vila-Chã et al., 2012; Pelliccia et al., 2018; Christou and O’Driscoll, 2020). The relative contribution of these adaptations to endurance exercise performance remains to be elucidated. Moreover, the few studies having investigated the cardiac determinants of long-distance endurance performance, such as marathon race, have focused on the left heart, essentially neglecting the potential role of right heart (Legaz Arrese et al., 2005, 2006a, 2006b).

The interplay of physiological determinants of endurance exercise performance may be particularly complex in long-distance running, since the anthropometric and body composition characteristics of athletes have an additional significance, due to the weight-bearing nature of running (Barandun et al., 2012; Tanda and Knechtle, 2015; Salinero et al., 2017). The impact of endurance exercise training on running performance appears to vary according to the distance of the race. Indeed, as running distance increases from middle-distance to long-distance races the impact of aerobic adaptations on running performance has been found to become increasingly important (Hill, 1999; Gastin, 2001; Duffield et al., 2005). However, performance in ultramarathon races has been demonstrated to be particularly influenced in a critical manner by strategies applied during the race about food, fluid and sodium intake (Stuempfle et al., 2011; Martinez et al., 2018). Thus, marathon race may represent the ideal model for the investigation of the role of endurance exercise adaptations in the determination of running performance in long-distance races.

Studies to date did not perform analysis of all possible physiological factors influencing the endurance exercise performance in active marathoners in an integrative manner. Uncovering the physiological determinants of marathon performance and their relative importance may prove invaluable for understanding the real nature of these changes, since it can be clarified whether they represent performance enhancing factors or simply reflect the chronic adaptations of endurance exercise training. Furthermore, the importance of such a study lies in the identification of possible modifiable physiological factors that if changed could enhance marathon performance. Although training characteristics, anthropometrics and spiroergometric parameters have been well-studied previously as determinants of marathon performance, the relevant role of cardiorespiratory and haematological adaptations needs further investigation (Tanda and Knechtle, 2015; Alvero-Cruz et al., 2020; Nikolaidis et al., 2021). Therefore, the aim of this study was to investigate, by means of anthropometry, echocardiography, cardiopulmonary exercise testing and blood analysis, the main anthropometric, cardiorespiratory and haematological factors that can determine marathon race performance in marathon runners.

## Materials and Methods

### Subjects

**FIGURE 1:**
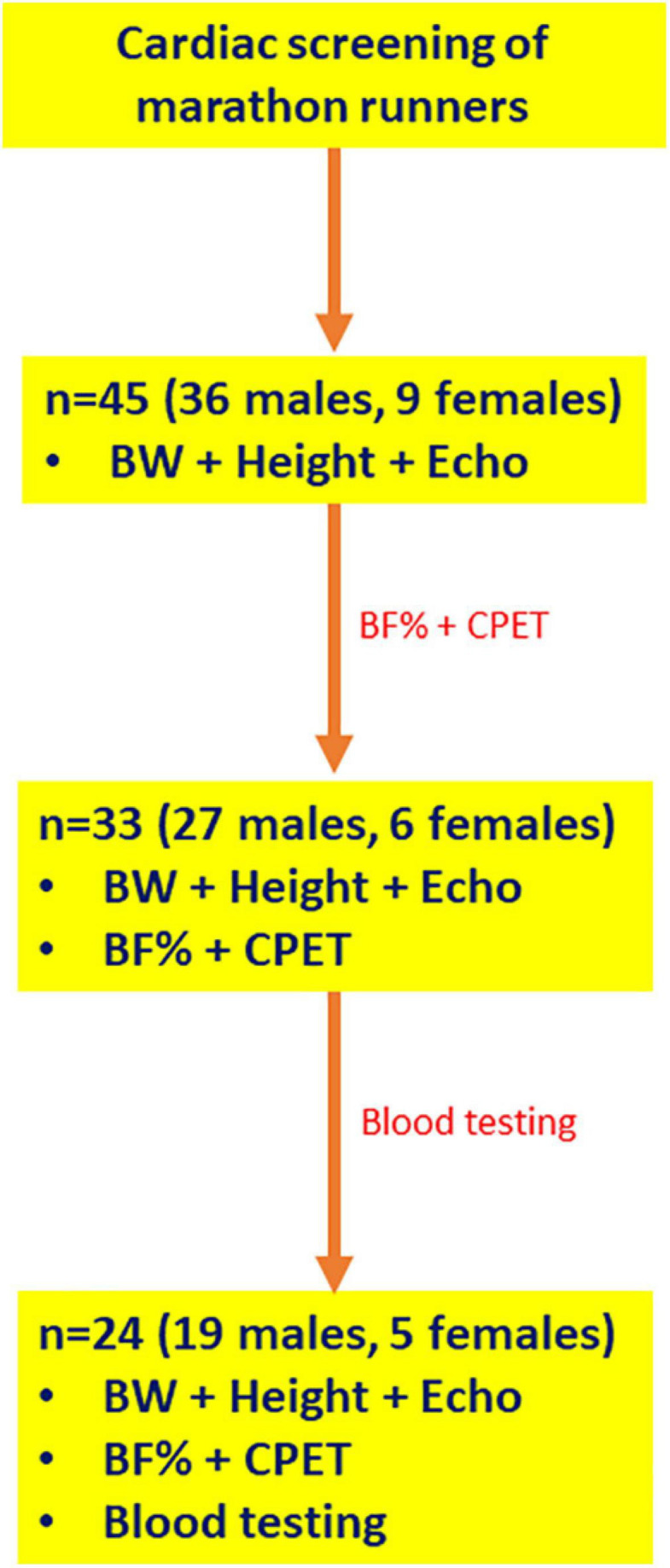
Flowchart showing the selection of study participants. BF%, body fat percentage; BW, body weight; CPET, cardiopulmonary exercise testing; Echo, transthoracic echocardiography.

The participants were consecutively recruited in the context of cardiac screening performed in the Laboratory of Sports Medicine of the Aristotle University of Thessaloniki in Greece in 2019 and 2020. Forty-five marathon runners who were training for a marathon race volunteered to participate in the study (Figure 1). The personal best marathon race time (MRT) within a time frame of one year before or after the evaluation of each athlete was selected as the parameter that best reflected the adaptations related to endurance exercise training. Inclusion criteria were: minimum running experience in long-distance races of one year and participation in at least one marathon race accredited by Association of International Marathons and Distance Races (AIMS)/International Association of Athletics Federations (IAAF) within a time frame of one year before or after the screening of each individual athlete. Exclusion criteria were: presence of any disease and use of performance enhancing drugs (based on history taking and physical examination for the detection of signs indicative of concealed use of performance enhancing drugs) (Christou et al., 2019).

A detailed medical and athletic history was taken, including information about the current training regimen and training age. All participants (36 males, 9 females) were subjected to measurement of height and body weight (BW) and transthoracic echocardiography. Thirty-three athletes (27 males, 6 females) consented to undergo assessment of body composition and cardiopulmonary exercise testing during screening. Twenty-four athletes (19 males, 5 females) admitted to be subjected to blood testing during screening. In total, 24 athletes (19 males and 5 females) underwent all measurements.

All participants gave a written informed consent. The study was conducted in accordance with the Declaration of Helsinki. The study protocol was approved by the Ethics Committee of Aristotle University of Thessaloniki (EC-3/2020). The trial was registered in ClinicalsTrials.gov (NCT04738877).

### Assessment of Anthropometrics

The athletes’ height and BW were measured. Percentage of body fat (BF%) was calculated using the formula of Durnin and Womersley based on four skinfold measurements (biceps, triceps, subscapula, and suprailium) (Durnin and Womersley, 1974; Chambers et al., 2014). For athletes with very low BF% (i.e., males: <12%, females: <15%) the formula of Jackson and Pollock was used based on three skinfold measurements (males: chest, abdomen and thigh, females: triceps, suprailium and thigh) (Jackson and Pollock, 1985; Sinning et al., 1985). Body fat mass (BFM) was calculated as BW × BF%/100 and lean body mass (LBM) as BW – BFM.

### Echocardiography

All echocardiographic images were acquired by two experienced cardiologists-ultrasonographers using commercially available ultrasound systems (Vivid I; GE Medical; Horten, Norway) with a 1.5- to 4-MHz phased-array transducer applying the same echo settings and following the same acquisition protocols. All studies were then analysed in a random order (to avoid bias) by the two cardiologists who had performed the acquisition of the images. A comprehensive assessment of the structure and function of the left and right heart was undertaken in accordance with the guidelines of European Association of Cardiovascular Imaging (Rudski et al., 2010; Lang et al., 2015; Nagueh et al., 2016). The ratio [MVE/Ea(s-l)] of the early diastolic transmitral flow velocity (MVE) to the average of septal and lateral early diastolic mitral annular velocity was used as an estimate of left ventricular (LV) filling pressures. Pulmonary vascular resistance was estimated using the formula: (TRVmax/RVOTTVI) × 10+0.16 (in Wood Units), where TRV_max_ is the peak velocity (in m/s) of the tricuspid valve regurgitant jet with continuous wave Doppler and RVOTTVI is the time-velocity integral in the right ventricular (RV) outflow tract.

### Cardiopulmonary Exercise Testing

Cardiopulmonary exercise testing was performed until exhaustion on a treadmill with the use of an ergometer (Ultima Series, Medgraphics, Minnesota, United States) applying BRUCE protocol. Specifically, the duration of each stage was 3 min with progressive increase in the grade and speed of the treadmill (Stage 1: 10% – 2.7 km/h, Stage 2: 12% – 4.0 km/h, Stage 3: 14% – 5.4 km/h, Stage 4: 16% – 6.7 km/h, Stage 5: 18% – 8.0 km/h, Stage 6: 20% – 8.8 km/h, Stage 7: 22% – 9.6 km/h) (Bruce et al., 1973). The athletes exercised under continuous 12-lead electrocardiographic monitoring. Blood pressure was measured every 3 min during exercise, as well as during the recovery period. After the termination of exercise, the participants remained during the recovery phase in a sitting position for 5 min under continuous electrocardiographic monitoring.

The following spiroergometric parameters were measured: maximum oxygen uptake (VO_2max_) during exercise and oxygen uptake at the first (VO_2_-VT1) and second (VO_2_-VT2) ventilatory threshold (Balady et al., 2010; Guazzi et al., 2012, 2018). Criteria for VO_2max_ were plateau in oxygen uptake despite an increase in workload, respiratory exchange ratio (RER) ≥1.10 and maximum heart rate ≥95% of the age-predicted value (Howley et al., 1995; Bassett and Howley, 2000). The detection of VT1 and VT2 was achieved with the combined use of three methods: V-slope, ventilatory equivalents and end-tidal gases. Oxygen pulse, which is the ratio of oxygen consumption to heart rate, was calculated (Balady et al., 2010). The oxygen pulse provides an estimate of LV stroke volume during exercise. Relative oxygen pulse was determined as the ratio of oxygen pulse to BW (Perim et al., 2011). We calculated the physiological dead space to tidal volume ratio (V_d_/V_t_) during exercise (Balady et al., 2010).

### Blood Measurements

Blood chemistry was performed with fresh blood samples using the Roche Cobas 6000 Chemistry Analyzer (Roche Diagnostics, Basel, CH, United States) the day that blood sampling was performed. Blood measurements included a full blood count, ferrous concentration, ferritin, vitamin B12, folic acid, sodium, potassium, calcium and magnesium. Taking into account that LBM represents an estimate of blood volume, the product of haemoglobin concentration ([Hb]) and LBM was used as a proxy measure for haemoglobin mass (Hbmass) (Retzlaff et al., 1969; Boer, 1984; Raes et al., 2006).

### Statistical Analysis

All statistical analyses were performed using the software IBM SPSS Statistics 23.0. Kolmogorov–Smirnov test was used to verify the normality of the distributions of the parameters of interest. Parameters with normal distribution were expressed as mean ± standard deviation and with skewed distribution as median (minimum–maximum). Mann–Whitney *U* test was performed for comparisons between two independent groups. The associations between the parameters of interest were assessed with Spearman’s correlation analysis. The strength of the correlation was considered strong, moderate and weak for values of Spearman’s correlation coefficient (rho) ≥0.70, 0.40–0.70 and <0.40 respectively (Akoglu, 2018). Multivariate linear regression analysis was used to identify the parameters with independent associations. Receiver-operating characteristic (ROC) curve analysis was used to evaluate whether the studied parameters could discriminate between athletes with MRT < 3:00:00 and the ones with MRT ≥ 3:00:00. The discriminatory ability was considered as fail, poor, fair, good and excellent for area under the curve (AUC) values of 0.5–0.6, 0.6–0.7, 0.7–0.8, 0.8–0.9 and 0.9–1.0 respectively. The relevant optimal cut-off values of these parameters were selected to conform with Youden’s index [J = max(sensitivity+specificity–1)]. A two-tailed *p* value <0.05 was considered statistically significant.

## Results

### Characteristics of the Athletes

Demographic, training and race performance characteristics of the athletes are shown in Table 1. The intervening time between study measurements and marathon race was 26 ± 18 weeks.

**TABLE 1:** Demographic, training, and race performance characteristics of the athletes.

	**All (*n* = 45)**	**Males (*n* = 36)**	**Females (*n* = 9)**
Race	Caucasians		
Sex (males/females)	36/9		
Age (years)	42 ± 10	42 ± 9	42 ± 10
Training age (years)	9 ± 7	18 ± 12	4 ± 3
Training volume (km/week)	70 (50–175)	70 (50–175)	60 (50–70)
MRT (h:min:sec)	4:02:53 ± 00:50:20	3:51:50 ± 00:45:41	4:43:53 ± 0:42:29
Professional/Amateur	7/38	7/29	0/9

*Data are expressed as mean ± standard deviation for normally distributed variables or median (minimum–maximum) for non-normal variables. Training age refers to long-distance running. Professional athletes were considered those with MRT < 3:00:00.*

*MRT, Marathon race time.*

MRT was faster in males compared to females (3:52:38 ± 0:47:14 vs 4:43:53 ± 0:42:29, *p* = 0.007). MRT correlated negatively with training age (rho = −0.530, *p* = 0.001) and training volume (rho = –0.741, *p* < 0.001), but not with age (rho = 0.208, *p* = 0.186).

### Anthropometrics and Body Composition

Mean height was 175 ± 7 cm, BW: 72.5 ± 10.3 kg, BF%: 17.5 ± 7.5%, BFM: 12.8 ± 6.0 kg and LBM: 60.3 ± 8.9 kg. Males were characterised by greater height (177 ± 6 vs 167 ± 6 cm, *p* < 0.001), BW (75.3 ± 9.5 vs 61.2 ± 3.7 kg, *p* < 0.001) and LBM (63.0 ± 6.0 vs 44.7 ± 5.8 kg, *p* < 0.001), but lower BF% (16.1 ± 6.9 vs 24.8 ± 7.6 %, *p* = 0.022) compared to females. There was no difference in BFM (12.5 ± 6.3 vs 14.6 ± 4.2 kg, *p* = 0.314) between males and females.

**FIGURE 2:**
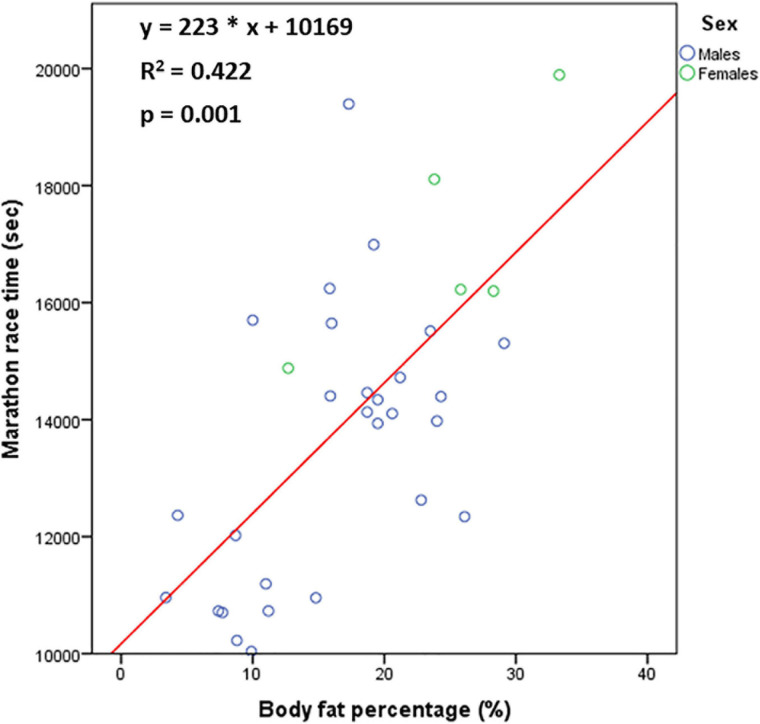
The relationship [*n* = 33 (27 males, 6 females)] between marathon race time and body fat percentage.

The MRT correlated positively with BF% (rho = 0.587, *p* = 0.001) and BFM (rho = 0.523, *p* = 0.003) and negatively with height (rho = −0.514, *p* < 0.001) and LBM (rho = −0.507, *p* = 0.005), but not with BW (rho = −0.064, *p* = 0.688) (Figure 2). The association between MRT and BF% remained even after adjustment for absolute values (i.e., in mL/min) of VO_2_-VT1, VO_2_-VT2 or VO_2_max. Among males, MRT correlated positively with BF% (rho = 0.568, *p* = 0.002) and BFM (rho = 0.571, *p* = 0.002), but not with BW, height and LBM.

### Parameters of Cardiopulmonary Exercise Testing

**FIGURE 3:**
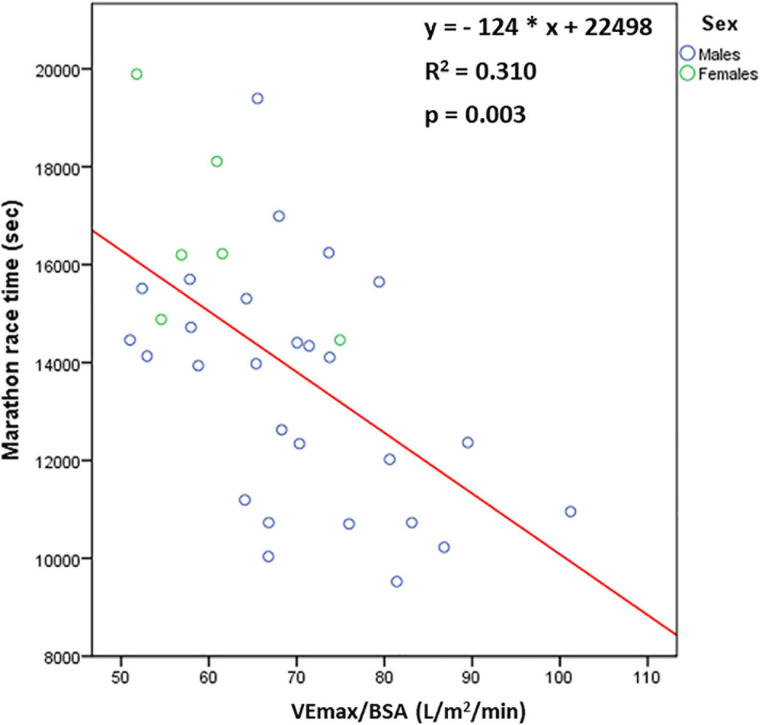
The relationship [*n* = 33 (27 males, 6 females)] between marathon race time and maximum minute ventilation indexed to body surface area (VEmax/BSA).

The values of parameters of cardiopulmonary exercise testing and their relationship with MRT are displayed in Table 2. The following parameters correlated negatively with MRT in both the total group of athletes and males: total exercise time, maximum tidal volume indexed to body surface area (Vtmax/BSA), maximum minute ventilation indexed to BSA (VEmax/BSA), VO_2_-VT1, VO_2_-VT2, VO_2_max and maximum relative oxygen pulse (Figure 3).

**TABLE 2:** Values of parameters of cardiopulmonary exercise testing and their relationship with marathon race time (MRT).

	**All (*n* = 33)**	**Males (*n* = 27)**	**Females (*n* = 6)**	**MRT**			
				**All (*n* = 33)**		**Males (*n* = 27)**	
				**rho**	***p* value**	**rho**	***p* value**
ExTime (min:sec)	17:12 ± 3:18	17:44 ± 3:29	15:08 ± 1:04	–0.583	<0.001	–0.507	0.010
HRmax (bpm)	177 ± 11	176 ± 12	181 ± 7	0.154	0.351	0.221	0.288
SBPpeak (mmHg)	190 (155–220)	200 (170–220)	175 (155–210)	–0.361	0.039	–0.007	0.976
DBPpeak (mmHg)	75 (60–85)	75 (60–85)	75 (60–80)	–0.258	0.129	–0.354	0.090
Vtmax (L)	2.705 ± 0.448	2.832 ± 0.360	2.133 ± 0.363	–0.340	0.061	–0.050	0.814
Vtmax/BSA (L/m^2^)	1.440 ± 0.214	1.479 ± 0.202	1.266 ± 0.190	–0.439	0.014	–0.398	0.044
RRmax (br/min)	48 ± 9	48 ± 9	48 ± 9	–0.238	0.182	–0.494	0.012
VEmax (L/min)	128.4 ± 23.5	134.3 ± 20.2	101.6 ± 19.1	–0.444	0.011	–0.309	0.117
VEmax/BSA (L/m^2^/min)	68.4 ± 11.9	70.3 ± 12.0	60.1 ± 8.2	–0.509	0.003	–0.463	0.017
(Vd/Vt)peak	0.20 ± 0.06	0.19 ± 0.06	0.22 ± 0.05	–0.109	0.551	–0.007	0.974
VO_2_-VT1 (mL/kg/min)	25.0 ± 7.8	26.1 ± 8.2	19.7 ± 2.1	–0.476	0.006	–0.436	0.029
VO_2_-VT2 (mL/kg/min)	43.7 ± 9.9	45.5 ± 10.0	35.4 ± 3.3	–0.704	<0.001	–0.725	<0.001
VO_2_max (mL/kg/min)	53.2 ± 9.7	54.9 ± 9.8	45.4 ± 4.1	–0.719	<0.001	–0.725	<0.001
Maximum oxygen pulse (mL/beat)	22 ± 5	24 ± 4	15 ± 2	–0.534	0.002	–0.347	0.076
Maximum relative oxygen pulse (mL/kg/beat)	0.30 ± 0.05	0.32 ± 0.05	0.25 ± 0.02	–0.834	<0.001	–0.823	<0.001
RERpeak	1.27 ± 0.19	1.23 ± 0.16	1.45 ± 0.21	0.337	0.055	0.227	0.256

*Data are expressed as mean ± standard deviation for normally distributed variables or median (minimum–maximum) for non-normal variables.*

*DBPpeak, diastolic blood pressure at peak exercise; ExTime, total exercise time; HRmax, maximum heart rate; RERpeak, peak respiratory exchange ratio; RRmax, maximum respiratory rate; SBPpeak, systolic blood pressure at peak exercise; Vd/Vt)peak, physiological dead space to tidal volume ratio at peak exercise; VEmax, maximum minute ventilation; VEmax/BSA, maximum minute ventilation indexed to body surface area; VO_2_max, body weight-indexed maximum oxygen uptake; VO_2_-VT1, body weight-indexed oxygen uptake at first ventilatory threshold; VO_2_-VT2, body weight-indexed oxygen uptake at second ventilatory threshold; Vtmax, maximum tidal volume; Vtmax/BSA, maximum tidal volume indexed to body surface area.*

The BSA was associated with both Vtmax (rho = 0.460, *p* = 0.009) and VEmax (rho = 0.384, *p* = 0.036).

### Echocardiographic Parameters

Table 3 shows the values of echocardiographic parameters and their relationship with MRT. The following parameters correlated positively with MRT in both the total group of athletes and males: average of septal and lateral late diastolic mitral annular velocity [MVA(s-l)], MVE/Ea(s-l) and systolic tricuspid annular velocity (TVSa). The following parameters correlated negatively with MRT in both the total group of athletes and males: mid-cavity (RVmid), and longitudinal (RVlon) RV diameter, and RV end-diastolic area (RVEDA).

**TABLE 3:** Values of echocardiographic parameters and their relationship with marathon race time (MRT).

	**All (*n* = 45)**	**Males (*n* = 36)**	**Females (*n* = 9)**	**MRT**			
				**All (*n* = 45)**		**Males (*n* = 36)**	
				**rho**	***p* value**	**rho**	***p* value**
LVIVSd (cm)	0.7 (0.5–1.0)	0.6 (0.5–1.0)	0.7 (0.5–0.8)	0.087	0.582	0.128	0.471
LVPWTd (cm)	0.7 (0.5–1.0)	0.6 (0.5–1.0)	0.7 (0.5–0.8)	0.053	0.741	0.168	0.343
LVIDd (cm)	5.0 ± 0.5	5.1 ± 0.4	4.6 ± 0.3	–0.240	0.121	–0.055	0.750
LVEDV (mL)	165 (107–284)	180 (131–284)	136 (107–181)	–0.400	0.007	–0.187	0.274
LVEDVi (mL/m^2^)	89 (64–151)	93 (67–151)	82 (64–99)	–0.382	0.010	–0.242	0.161
LVmass (g)	107 ± 26	111 ± 25	93 ± 23	–0.270	0.083	–0.086	0.634
LAV (mL)	46 ± 16	48 ± 17	38 ± 10	–0.292	0.052	–0.181	0.290
LVEF (%)	51 ± 6	50 ± 6	53 ± 3	0.351	0.020	0.179	0.295
SV (mL)	90 ± 25	94 ± 26	73 ± 10	–0.214	0.158	–0.046	0.793
MVE/A	1.8 (0.8–3.7)	1.7 (0.9–3.7)	1.9 (0.8–2.4)	–0.179	0.250	–0.184	0.306
MVEa(s-l) (m/sec)	0.15 ± 0.04	0.15 ± 0.04	0.16 ± 0.03	–0.054	0.743	–0.417	0.016
MVAa(s-l) (m/sec)	0.10 ± 0.02	0.10 ± 0.02	0.10 ± 0.01	0.583	<0.001	0.667	<0.001
MVSa(s-l) (m/sec)	0.10 ± 0.02	0.10 ± 0.02	0.10 ± 0.01	–0.142	0.364	–0.137	0.441
MVE/Ea(s-l)	5.3 ± 1.2	5.3 ± 1.3	5.2 ± 0.9	0.352	0.020	0.411	0.014
RVbas (cm)	4.2 ± 0.8	4.4 ± 0.8	3.6 ± 0.5	–0.384	0.012	–0.290	0.101
RVmid (cm)	3.6 ± 0.7	3.7 ± 0.7	2.9 ± 0.6	–0.709	<0.001	–0.585	0.001
RVlon (cm)	8.0 ± 0.9	8.1 ± 0	7.7 ± 1.1	–0.461	0.004	–0.436	0.016
RVOTprox (cm)	3.4 ± 0.6	3.6 ± 0.5	2.7 ± 0.5	–0.542	<0.001	–0.319	0.066
RVEDA (cm^2^)	26.6 ± 5.8	27.9 ± 5.2	21.3 ± 4.7	–0.716	<0.001	–0.717	<0.001
RAA (cm^2^)	17.4 ± 5.6	18.5 ± 5.5	13.0 ± 3.0	–0.379	0.011	–0.070	0.683
RVFAC (%)	41 ± 9	43 ± 7	36.7 ± 12.1	–0.124	0.470	–0.044	0.821
TVE/A	1.8 ± 0.5	1.8 ± 0.5	2.0 ± 0.6	–0.258	0.123	–0.268	0.138
TVEa (m/sec)	0.15 ± 0.04	0.15 ± 0.04	0.17 ± 0.03	0.197	0.200	0.146	0.416
TVAa (m/sec)	0.14 ± 0.04	0.14 ± 0.04	0.16 ± 0.05	0.466	0.002	0.244	0.178
TVSa (m/sec)	0.16 ± 0.03	0.16 ± 0.03	0.15 ± 0.02	0.329	0.031	0.426	0.014
TVE/Ea	3.6 ± 1.1	3.7 ± 1.2	3.0 ± 0.1	–0.103	0.533	–0.011	0.949
TAPSE (cm)	2.7 ± 0.4	2.7 ± 0.5	2.7 ± 0.3	–0.237	0.131	–0.114	0.516
PASP (mmHg)	21 ± 8	20 ± 9	21 ± 8	0.029	0.886	–0.168	0.456
PVR (Wood)	1.08 ± 0.33	1.05 ± 0.32	1.21 ± 0.37	0.038	0.840	–0.022	0.919

*Data are expressed as mean ± standard deviation for normally distributed variables or median (minimum–maximum) for non-normal variables.*

*LAV, left atrial end-systolic volume; LVEDV, left ventricular end-diastolic volume; LVEDVi, left ventricular end-diastolic volume indexed to body surface area; LVEF, left ventricular ejection fraction; LVIDd, left ventricular end-diastolic internal diameter; LVIVSd, left ventricular interventricular septum thickness at end-diastole; LVmass, left ventricular mass; LVPWTd, left ventricular posterior wall thickness at end-diastole; MVA, late diastolic transmitral flow velocity; MVA(s-l), average of septal and lateral late diastolic mitral annular velocity; MVE, early diastolic transmitral flow velocity; MVE/A, ratio of early to late diastolic transmitral flow velocity; MVEa(s-l), average of septal and lateral early diastolic mitral annular velocity; MVE/Ea(s-l), ratio of the early diastolic transmitral flow velocity to the average of septal and lateral early diastolic mitral annular velocity; MVSa(s-l), average of septal and lateral systolic mitral annular velocity; PASP, pulmonary artery systolic pressure; PVR, pulmonary vascular resistance; RAA, right atrial end-systolic area; RVbas, basal right ventricular diameter; RVEDA, right ventricular end-diastolic area; RVFAC, right ventricular fractional area change; RVlon, right ventricular longitudinal diameter; RVmid, right ventricular mid-cavity diameter; RVOTprox, proximal right ventricular outflow tract diameter; SV, stroke volume; TAPSE, tricuspid annular plane systolic excursion; TVA, late diastolic transtricuspid flow velocity; TVAa, late diastolic tricuspid annular velocity; TVE, early diastolic transtricuspid flow velocity; TVEa, early diastolic tricuspid annular velocity; TVE/A, ratio of early to late diastolic transtricuspid flow velocity; TVE/Ea,ratio of early diastolic transtricuspid flow velocity to early diastolic tricuspid annular velocity; TVSa, systolic tricuspid annular velocity.*

Among these echocardiographic parameters that correlated significantly with MRT, we aimed to explore which of them were associated with the spiroergometric determinants of MRT reflecting greater functional capacity (i.e., VO_2_max) and greater stroke volume during exercise (i.e., maximum relative oxygen pulse). Among the echocardiographic parameters, VO_2_max correlated positively with RVmid (rho = 0.490, *p* = 0.009) and RVEDA (rho = 0.650, *p* < 0.001) and negatively with MVA(s-l) (rho = −0.566, *p* = 0.001). Maximum relative oxygen pulse correlated positively with RVmid (rho = 0.642, *p* < 0.001) and RVEDA (rho = 0.764, *p* < 0.001) and negatively with MVA(s-l) (rho = −0.373, *p* = 0.039).

We attempted to elucidate whether body size contributed significantly to the variation of LV and RV size, since the strength of the relationship of BSA with these parameters may weaken the predictive power of the studied parameters to determine marathon performance. Thus, BSA was associated with LVEDV (rho = 0.594, *p* < 0.001), but not with RVEDA (rho = 0.245, *p* = 0.144).

**FIGURE 4:**
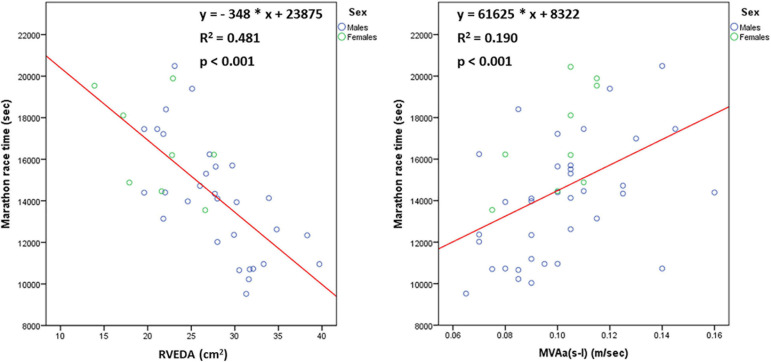
The relationship [*n* = 45 (36 males, 9 females)] of marathon race time with right ventricular end-diastolic area (RVEDA) and average of septal and lateral late diastolic mitral annular velocity [MVA(s-l)].

When we applied multivariate linear regression analysis in the total group of athletes with MRT as dependent variable and MVA(s-l) and RVEDA as independent variables, both RVEDA (standardised β coefficient = −0.600, *p* < 0.001) and MVAs-l (standardised β coefficient = 0.266, *p* = 0.047) were independent predictors of MRT (adjusted R^2^ = 0.538, *p* < 0.001 for the model), whereas RVEDA was the only independent predictor of MRT in males (Figure 4).

### Parameters of Blood Test

**FIGURE 5:**
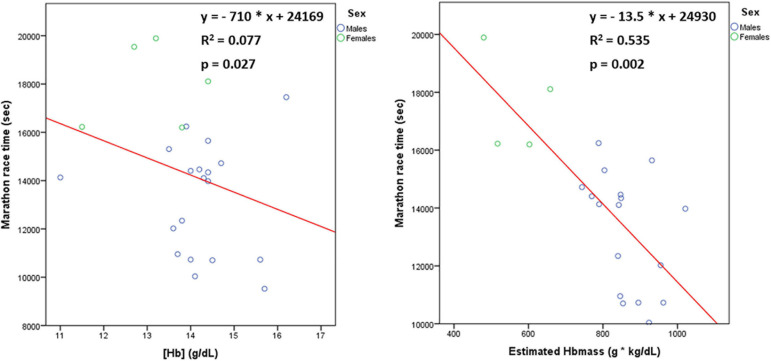
The relationship [*n* = 24 (19 males, 5 females)] of marathon race time with haemoglobin concentration ([Hb]) and estimated haemoglobin mass (Hbmass).

The [Hb] was 14.0 ± 1.1 g/dL, haematocrit: 41.5 ± 3.3%, ferrous concentration: 110 ± 37 μg/dL, ferritin: 82 ± 62 ng/mL, vitamin B12: 390 ± 112 pg/mL and folic acid: 8.7 ± 2.8 ng/mL. Males tended to have greater [Hb] (14.2 ± 1.1 vs 13.1 ± 1.1 g/dL, *p* = 0.053), ferritin (94 ± 65 vs 41 ± 23 ng/mL, *p* = 0.058) and vitamin B12 (414 ± 119 vs 322 ± 56 pg/mL, *p* = 0.070), compared to females, whereas there was no difference in haematocrit (42.1 ± 3.0 vs 39.4 ± 4.0 %, *p* = 0.120), ferrous concentration (111 ± 38 vs 106 ± 37 μg/dL, *p* = 0.880) and folic acid (8.6 ± 3.1 vs 9.1 ± 1.8 ng/mL, *p* = 0.446) between males and females. With regard to serum electrolytes, sodium was 140 ± 1 mmol/L, potassium: 4.4 ± 0.3 mmmol/L, calcium: 9.5 ± 0.4 mg/dL and magnesium: 2.1 ± 0.2 mg/dL. Among these parameters of blood test, MRT correlated negatively with [Hb] (rho = −0.471, *p* = 0.027) and vitamin B12 (rho = −0.657, *p* = 0.003) (Figure 5). Among males, MRT correlated significantly only with vitamin B12 (rho = −0.610, *p* = 0.027), but not with [Hb]. All athletes with MRT < 3:00:00 were characterised by vitamin B12 > 400 pg/mL. MRT was strongly associated with estimated Hbmass (rho = −0.680, *p* = 0.002) (Figure 5). The relationship between MRT and estimated Hbmass was also significant in males (rho = −0.559, *p* = 0.024).

### Integrative Evaluation of the Physiological Determinants of MRT

The above-mentioned results indicate that the main physiological determinants of MRT were the following. Anthropometric: BF%, cardiac: RVEDA, respiratory: VEmax/BSA and haematological: Hbmass. All these parameters remained significant predictors of MRT after adjustment for sex or professional status or whether blood measurements were performed or not. After performing multivariate linear regression analysis with MRT as dependent variable and BF% (standardised β = 0.501, *p* = 0.021), RVEDA (standardised β = −0.633, *p* = 0.003), VEmax/BSA (standardised β = 0.266, *p* = 0.303) and Hbmass (standardised β = −0.308, *p* = 0.066) as independent variables, only BF% and RVEDA were significant independent predictors of MRT (adjusted R^2^ = 0.796, *p* < 0.001 for the model).

**FIGURE 6:**
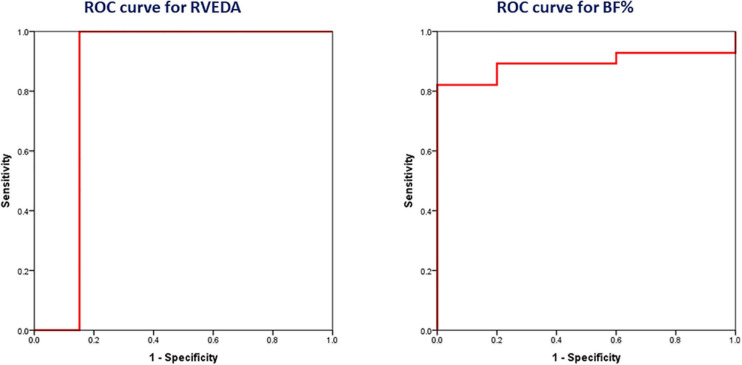
Receiver-operating characteristic (ROC) curves showing the ability of right ventricular end-diastolic area (RVEDA) [*n* = 45 (36 males, 9 females)] and body fat percentage (BF%) [*n* = 33 (27 males, 6 females)] to discriminate between athletes with marathon race time (MRT) < 3:00:00 and the ones with MRT ≥ 3:00:00.

After applying ROC curve analysis to discriminate between athletes with MRT < 3:00:00 and the ones with MRT ≥ 3:00:00, the AUC value was 0.848 (95% CI: 0.726–0.971) for RVEDA (*p* = 0.013) and 0.893 (95% CI: 0.783–1.000) for BF% (*p* = 0.006), indicating good discriminatory ability for both parameters (Figure 6). We selected the optimal cut-off values of RVEDA = 30.4 cm^2^, and BF% = 12.0% to conform with Youden’s index.

## Discussion

The present study showed that the main anthropometric determinant of marathon performance was BF%, via the impact on running economy and the principal cardiac adaptation that could predict a better marathon performance was right ventricular enlargement, through the increase in stroke volume during exercise. Maximum minute ventilation during exercise was found to be a weaker respiratory predictor of marathon performance, mainly attributed to regulation of tidal volume. Estimated haemoglobin mass was demonstrated to be a much stronger predictor of marathon performance than haemoglobin concentration.

### Anthropometric Determinants of MRT

A low BF% appeared to be the most powerful anthropometric determinant of better marathon performance, as previously shown, with values lower than 12% adequately predicting a MRT < 3:00:00 (Barandun et al., 2012; Tanda and Knechtle, 2015; Salinero et al., 2017). The fact that the association between MRT and BF% remained even after adjustment for the absolute values of VO_2_-VT1, VO_2_-VT2 or VO_2_max indicates that a low BF% is possibly advantageous for marathon performance, due to a greater mechanical efficacy of energy utilisation (Bassett and Howley, 2000). In this respect, a low BF% possibly determines a better running economy. Notably, the weight-bearing nature of running was reflected by the existence of relationship of MRT with BF%, but not with BW, highlighting the great influential role of body composition on running economy, as opposed to a simple consideration of BW.

### Cardiac Determinants of MRT

The present study showed that the cardiac determinants of better marathon performance were cardiac chamber enlargement and improved ventricular diastolic function, with the former being more important. Notably, among cardiac chamber indices, RV enlargement was the main cardiac determinant of marathon performance, with values greater than 30 cm^2^ adequately predicting a MRT < 3:00:00, highlighting the high importance of adequate pulmonary perfusion to support prolonged endurance exercise. The RVEDA was demonstrated to be a much stronger predictor of MRT than LVEDV. Indeed, considering that LVEDV was found to be influenced more strongly by BSA compared to RVEDA, RV enlargement may reflect more accurately cardiac adaptations of endurance exercise training in marathon runners, whereas LV enlargement may be caused not only by endurance exercise training, but large body habitus as well. The association between MRT and RVEDA appears to be mainly attributed to an increase in RVmid, indicating the importance of transverse RV remodeling in marathon performance. The relationship of the cardiac determinants of MRT with maximum oxygen pulse during exercise implies that the underlying physiological mechanism is possibly the upregulation of stroke volume during exercise (Perim et al., 2011; Lang et al., 2015). In this respect, RV enlargement in marathon runners possibly represents a useful myocardial adaptation to endurance exercise training, rather than a sign of RV dysfunction and thus the benign nature of this adaptation should be distinguished from arrhythmogenic right ventricular cardiomyopathy (D’Ascenzi et al., 2018).

Only two studies have investigated the association of echocardiographic parameters with marathon performance so far (Legaz Arrese et al., 2005, 2006b). Both these studies evaluated a small number of elite athletes and investigated only the relationship of MRT with LV end-diastolic internal diameter, essentially neglecting the relevant role of RV (Legaz Arrese et al., 2005, 2006b).

### Respiratory Determinants of MRT

The current study demonstrated for the first time that the principal respiratory determinant of better marathon performance was increased VEmax during exercise, mainly attributed to increased Vtmax during exercise (Mahler et al., 1982). Consistently, increased both VEmax and Vtmax during exercise have been reported in endurance athletes compared to nonathletes (Kippelen et al., 2005; Layton et al., 2011). In this respect, deep breathing at peak exercise may represent a more efficient ventilatory pattern in athletes, resulting in decreased V_d_/V_t_ and enhanced alveolar ventilation, as opposed to rapid sallow breathing at peak exercise (Lucía et al., 1999).

### Haematological Determinants of MRT

Although high [Hb] was found to correlate with better marathon performance, the association of MRT with estimated Hbmass was much stronger, highlighting the importance of haemoglobin mass, rather than haemoglobin concentration, to determine the oxygen capacity of blood (Prommer et al., 2010; Otto et al., 2013, 2017). Importantly, endurance exercise training has been reported to induce expansion of plasma volume, that can result in a small drop of [Hb] (Convertino et al., 1980; Heinicke et al., 2001; Otto et al., 2013). Therefore, simple consideration of [Hb] in endurance athletes may lead to underestimation of oxygen capacity of blood and subsequently could create unreasonable wariness about the performance decreasing effect of suboptimal [Hb] levels. This approach to assess the haematological profile of marathon runners through the estimation of haemoglobin mass, as the product of [Hb] and LBM, appears to be cheaper, easier and more convenient than the direct measurement of haemoglobin mass with the optimised CO-rebreathing method of Schmidt and Prommer and possibly allows a more accurate assessment of the oxygen capacity of blood compared to [Hb] (Schmidt and Prommer, 2005). Future studies could try to validate the accuracy of haemoglobin mass estimation with reference to the direct measurement of haemoglobin mass.

Furthermore, the present study showed for the first time that a high serum concentration of vitamin B12 was associated with better marathon performance. Although it can be reasonably assumed that high serum levels of vitamin B12 can be ergogenic through the stimulation of erythropoiesis, we cannot exclude the possibility that increased circulating vitamin B12 may reflect higher intake of supplements containing vitamin B12 by the faster runners, who are commonly more ambitious and overconsume ergogenic supplements (Krzywański et al., 2020). In accordance with the previously reported stimulating effect of circulating vitamin B12 > 400 pg/mL on erythropoiesis in athletes, we demonstrated that circulating B12 > 400 pg/mL was associated with MRT < 3:00:00 (Krzywański et al., 2020). Further randomised trials are needed to confirm whether exogenous supplementation of marathon runners with vitamin B12 can improve marathon performance.

### Study Strengths and Limitations

Strengths of this study include firstly the fact that the physiological determinants of marathon performance were investigated in an integrative manner, taking into account all their potential interactions. This holistic methodological approach appears to be particularly important to detect independent determinants of marathon performance, since there are many interrelated correlates of marathon performance. Secondly, taking into account that our study population included runners with a wide range of MRT, the findings of the present study can be applicable to the full spectrum of marathon performance, extending from amateur runners to elite athletes. Moreover, the personal best MRT within a time frame of one year before or after the evaluation of each athlete was selected as the parameter that best reflected the adaptations related to endurance exercise training, rather than marathon performance in a specific marathon race that may underestimate the full potential of a marathon runner in case of suboptimal performance in this race for various reasons Furthermore, we diligently evaluated for the first time the role of right heart structure and function to determine marathon performance.

The results of our study should be interpreted in light of some limitations. Firstly, the sample size was not large enough, implying the need for further studies to confirm the results of the present study. Secondly, males predominated over females. Besides, we performed correlation analysis in the total study population and separately in males, but not separately in females, due to their small number, that would not allow correct statistical analysis. Despite that, we demonstrated that the physiological determinants of marathon performance were the same after adjustment for sex, indicating that the results of the study may be applicable to both males and females. Additionally, although the number of athletes analysed was not the same for all analyses, the ratio of males/females included in each analysis was similar (i.e., 4/1), implying a uniform representation of the two sexes in all analyses. Further studies are needed to confirm the results of this study in a greater number of female marathon runners. Thirdly, the current study included a small number of professional athletes, though the main results were verified even after adjustment for professional status. Fourthly, it was not feasible to obtain blood samples from all the studied participants due to noncompliance. However, the results of this study remained unaltered after adjustment for whether blood measurements were performed or not. Moreover, a major limitation of the current study is that the potential of study measurements to reflect MRT may decrease with increasing length of intervening time between study measurements and marathon race (Coyle, 2005). Although the predictive ability of the measurements performed in the phase of detraining may be profoundly attenuated, all the athletes of this study were evaluated during the training phase of preparation for a marathon race (Pedlar et al., 2018). Finally, the present study focused on the investigation of central factors (i.e., cardiorespiratory and haematological factors) of VO_2_max as physiological determinants of marathon performance, without investigating the peripheral factors of VO_2_max, reflecting metabolic adaptations in skeletal muscle (Bassett and Howley, 2000).

## Conclusion

In conclusion, the main physiological determinants of better marathon performance appear to be low BF% and RV enlargement. Upregulation of both maximum minute ventilation during exercise and haemoglobin mass may have a weaker effect to enhance marathon performance. Further well-designed studies are needed to investigate whether the incorporation of these parameters in the existing methods used to assess serially the endurance capacity of marathon runners during the process of training periodisation can improve the management of these athletes.

## Data Availability Statement

The raw data supporting the conclusions of this article will be made available by the authors, without undue reservation.

## Ethics Statement

The studies involving human participants were reviewed and approved by Ethics Committee of Aristotle University of Thessaloniki. The patients/participants provided their written informed consent to participate in this study.

## Author Contributions

All authors listed have made a substantial, direct and intellectual contribution to the work, and approved it for publication.

## Conflict of Interest

The authors declare that the research was conducted in the absence of any commercial or financial relationships that could be construed as a potential conflict of interest.

## Publisher’s Note

All claims expressed in this article are solely those of the authors and do not necessarily represent those of their affiliated organizations, or those of the publisher, the editors and the reviewers. Any product that may be evaluated in this article, or claim that may be made by its manufacturer, is not guaranteed or endorsed by the publisher.
